# Comparison of different polar compounds-induced cytotoxicity in human hepatocellular carcinoma HepG2 cells

**DOI:** 10.1186/s12944-016-0201-z

**Published:** 2016-02-16

**Authors:** Jinwei Li, Xiaodan Li, Wenci Cai, Yuanfa Liu

**Affiliations:** State Key Laboratory of Food Science and Technology, Synergetic Innovation Center of Food Safety and Nutrition, School of Food Science and Technology, Jiangnan University, Wuxi, 214122 P.R. China; School of Food Science and Technology, Jiangnan University, 214122 Wuxi, Jiangsu Province China

**Keywords:** TGO, TGD, ox-TG, HepG2 cells, Apoptosis

## Abstract

Total polar compounds (TPC) formed during successive frying have the negative healthy effects. However, little researches focused on the cytotoxicity of different parts of TPC. The present study was carried out to elucidate the different polar compounds-induced apoptosis in human hepatocellular carcinoma (HCC) HepG2 cells. The polar compounds of frying oil named oxidized triglycerides oligo (TGO), oxidized triglycerides dimer (TGD), and oxidize triglycerides (ox-TG) were isolated and collected *via* HPLC. MTT assay was selected to determine the cell viability, and apoptosis rate of the cells was analyzed with the help of flow cytometry. The results indicated that TGO, TGD, or ox-TG could suppress the proliferation of HepG2 cells and improve the cell apoptosis in the concentration- and time- dependent manner. Different polar compounds have the different activity of cytotoxicity and apoptosis (*p* < 0.05), and ox-TG was the most serious deleterious on HepG2 cell viability and apoptotic, followed by TGO and TGD. At the concentration of 2.00 mg/ml and incubated for 48 h, the cell inhibition rate and apoptotic rate of HepG2 induced by ox-TG could reach 23.0 % and 16.05 %, respectively. Cell cycle analysis showed that the inhibition of TGO, TGD, and ox-TG on HepG2 cells mainly occurred in S phase.

## Introduction

Total polar compounds (TPC), which formed during processing and storage of fats and oils, have raised a great concern because of their adverse healthy effects. Heat is one of the risk factors contributing to the origination of TPC in technologic processes, such as vegetable oils refining, frying and cooking. It is well known that frying oils being used continuously at high temperatures might be subjected to thermal oxidation, polymerization, and hydrolysis in the presence of oxygen and water which is from the food being fried. Subsequently, a wide variety of chemical reactions occur, and these reactions could lead to the formation of kinds of compounds with high molecular and polarity, for instance oxidize triglycerides (ox-TG), oxidized triglycerides dimer (TGD), oxidized triglycerides oligo (TGO), diacylglycerol and some free fatty acids.

These polar compounds and polymeric triacylglycerides produced from degraded frying oils might have an adverse effect on health [1]. An early investigation indicated that it was highly possible that frying oils induce growth retardation, diarrhea, seborrhea, dermatitis, and hair loss in animals [2, 3]. Nwanguma et al. (1999) reported that the experimental rats fed with a thermally oxidized corn oil diet showed significantly higher relative liver weights than the control rats [4]. Liu et al. (2010) also reported that the rats fed with frying oil for 30 days caused gene mutations and chromosomal aberrations [5]. Billek indicated that feeding rats with polar compounds for 18 months resulted in lower body weight gain, an increase of liver and kidney weight, and a rise of serum glutamic pyruvic transaminase and glutamic oxaloacetic transaminase levels by 20 % approximately [6]. Therefore, many European countries, namely Spain, Portugal, France, Germany, Belgium, Switzerland, Italy, and the Netherlands, have established regulations to monitor the quality of used frying oils in restaurants based on the percentage of TPC. Maximum values ranging from 24 to 27 % by weight TPC designate when oil is unfit for use [7, 8].

TGO, TGD, and ox-TG are derived from oxidation of oils, and they composed most of the non-volatiles in TPC [9, 10]. TGO, TGD, and ox-TG play a vital role in oxidation process. However, former studies focused more on the negative effects of frying oils or TPC on health instead of TGO, TGD, or ox-TG. Moreover, the evaluations of TGO, TGD, or ox-TG-induced effects are controversial. Billek elucidated that TGO, TGD, or ox-TG was harmless because of the low resorption rate [5]. Other studies indicated that the resorption rate of triglyceride polymer (TGP) and ox-TG was high enough to induce toxic effects on animals [11, 12]. Nonetheless, more studies need to be done to illustrate the toxicity of TGO, TGD, or ox-TG developed during frying process.

HepG2 cells were derived from the livers of patients with hepatocellular carcinoma. These cells are comparable to normal hepatocytes considering the expression of specific enzymes and the enzyme activities [13–16]. Thus, HepG2 cells can be used to elucidate DNA damage caused by certain compounds, and it is a selectable model to perform the metabolic process. In the present study, the effects of TGO, TGD, or ox-TG on cell proliferation and apoptosis were determined, and the cytotoxicity of TGO, TGD, or ox-TG on HepG2 cells was analyzed.

## Materials and methods

### Chemicals

Frying oils was provided by Yihai Kerry (Shanghai, China). Silicon for chromatography was from Qingdao Haiyang Chemical Co., Ltd (Qingdao, Shandong, China). HepG2 cells were provided by School of Medicine & Pharmaceutics, Jiangnan University (Wuxi, Jiangsu, China). MTT [3-(4,5-dimethylthiazol-2-yl)-2,5- diphenytetrazolium-bromide] and DMSO (dimethyl sulfoxide) were purchased from Sigma (St. Louis, MO, USA). Hoechst 33258 Kit, Trypsin-EDTA Solution, Cell Cycle and Apoptosis Analysis Kit were from Beyotime (Shanghai, China). Fetal calf serum (FCS) and RPMI1640 were purchased from Gibco (Gaithersburg, MD, USA).

### Isolation and purification of TGD, TGD and ox-TG

TPC was prepared from recycled frying oil. Briefly, the frying oil was obtained from Yihai Kerry, Shanghai. According to AOCS Official Method Cd 20-91 [17], the chromatographic glass column with 20 mm internal diameter and 500 mm length, and 25 g of silica gel (containing 5 % water) was applied. The elution solvent was petroleum ether/diethyl ether (87:13, v/v). 2.5 g of heated frying oil being dissolved in the elution solvent (125 mg/mL) was introduced onto a glass column filled with silica gel (Branch of Qingdao Haiyang Chemical Co., Ltd) and elution solvent slurry. The non-polar compounds were eluted with 250 mL of the elution solvent, and then the polar fraction was eluted with 150 mL of diethyl ether. TPM was concentrated in a rotary evaporator under reduced pressure at 45 °C, the efficacy of which was determined by thin layer chromatography. TPM was dissolved with tetrahydrofuran to reach a final concentration of 6 mg/mL. Five milliliters of 6 mg/mL TPM tetrahydrofuran solution was filtrated through a filter membrane (0.22 μm, organic phase). Then, TPM was subjected to chromatography on a preparation type high-pressure silica chromatography column (30 * 250 mm, 5 μm, 100 Å, Bonna-Agela Technologies, Tianjin, China). Five peaks would be obtained. The conditions were as follows: flow rate was 1.0 ml/min, and the column temperature was held at 25 °C, injection volume was 10 μl, the gradient program consisted of 0–10 min 30 % (v/v) B (dichloromethane) and 70 % (v/v) A (hexane), 10–30 min 85 % (v/v) B and 15 % (v/v) A. The evaporative light detector spray temperature was set to 30 °C and the evaporation temperature was set to 42 °C. Nitrogen gas flow of 1 L/min was applied. TGD, TGD and ox-TG were collected respectively and they were concentrated in rotary evaporator evaporation.

### Cell culture

The human hepatocellular carcinoma cell line HepG2 (HB‐8065) was obtained from American Type Culture Collection (Manassas, VA, USA). The HepG2 cells were routinely grown in RPM1640 medium (52400–041, Gibco, USA) supplemented with 10 % heat-inactivated fetal calf serum (FCS, Gibco, USA) and 100 U/ml penicillin/streptomycin. The cells were incubated at 37 °C in a humidified atmosphere containing 5 % CO_2_. 1 ml trypsin digestion (0.02 % EDTA,0.25 % trypsin) was added when the number of cells achieved 80–90 %, and then the cells were placed at room temperature for 2 min. Then, 2 ml culture medium was added and was mixed gently to suspend the cells when the cells became round under microscope. The cells should be passaged every 2–3 days by 1/3 ratio. All the cells used in the experiments were in logarithmic phase.

### Measurement of cell viability

HepG2 cells (5 × 10^4^ cells/ml) were planted in 96-well microplates, and then the cells were treated with TGO, TGD, or ox-TG at various concentrations of 0.05, 0.25, 0.5, 1.0 and 2.0 mg/mL for 24, 48 and 72 h. Cell viability was determined by MTT assay [18]. In brief, Ten microliter of 5 mg/mL MTT solution in phosphate buffered-saline (PBS) was added into each well, and the plates were incubated at 37 °C for 4 h. One hundred microliter dimethyl sulfoxide (DMSO) was pipetted into each well after 4 h incubation. The absorbance of each well was then measured at 570 nm using a microplate reader (MK3; Thermo). Assuming 100 % survival for the negative control, the survival rates of various samples were calculated as follows:

$$ \mathrm{Inhibition}\ \mathrm{rate} = \left[1-\mathrm{O}\mathrm{D}\ \left(\mathrm{treated}\ \mathrm{sample}\right)/\mathrm{O}\mathrm{D}\ \left(\mathrm{blank}\ \mathrm{group}\right)\right]\times 100\ \% $$

### Cell cycle analysis

1 ml cells (10^6^ cells/ml) were seeded in 6-well plates and treated with different TGO, TGD, or ox-TG concentrations. After 24 and 48 h, the cells were collected and suspended in centrifuge tube, and then the cells were stained with 0.5 ml propidium iodide (PI). Being incubated 30 min at 37 °C in the dark, apoptosis of the cells would be examined by FACS Calibur flow cytometry (Becton Dickson, USA) at an excitation wavelength of 480 nm.

## Results and discussion

### The inhibition rate assessment of TGO, TGD, or ox-TG on HepG2 cells

**Fig. 1:**
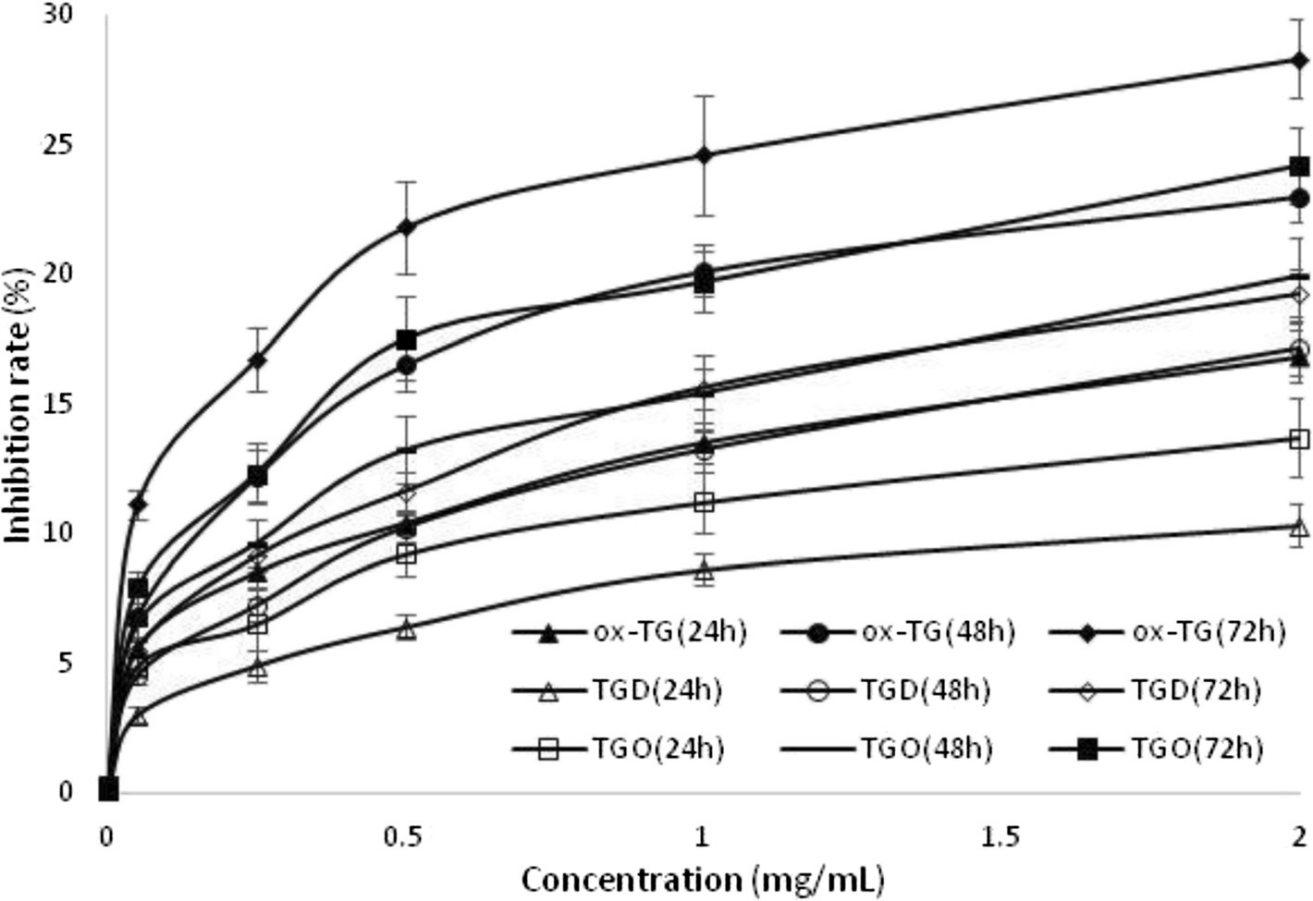
The inhibition rate of TGO, TGD, and ox-TG on HepG2 cells

The effects of TGO, TGD, or ox-TG on HepG2 cells viability were investigated with different time and concentrations (Fig. 1). The inhibition rate of TGO, TGD, and ox-TG on HepG2 cells was significantly different (*p <* 0.05) from each other, and their concentrations and incubation time significantly affected the cells viability (*p <* 0.05). When HepG2 cells were exposed to TGO, TGD and ox-TG at 0.25 mg/ml for 48 h, the inhibition rate of TGO, TGD, and ox-TG on the cells were 9.6 %, 7.2 %, and 12.2 %, respectively, which were 13.2 %, 10.2 %, and 16.5 % with a concentration of 0.5 mg/ml. The inhibition activity of TGO, TGD, and ox-TG on HepG2 cells decreased in the order of ox-TG > TGO > TGD. Compared to TGO and TGD, ox-TG with a higher polarity is more active, and it is more easily to react with oxygen [19]. The inhibition rate on the cells increased in a concentration-dependent manner with increasing concentrations of TGO, TGD, and ox-TG. Furthermore, at the relatively low concentration range of 0–0.5 mg/mL, the inhibition rate of TGO, TGD, and ox-TG on the cells increased significantly with increasing concentrations. The inhibition activity of TGO, TGD, or ox-TG on HepG2 cells also enhanced with an increased treated time. Exposed to HepG2 cells for 24 h, 48 h and 72 h to 0.5 mg/ml ox-TG resulted in the inhibition rate on the cells were 10.4 %, 16.5 % and 21.8 %, respectively, which was significantly different from the control (*P* < 0.05). When the concentration improved to 2.0 mg/ml, the inhibition rate could reach 16.8 %. 23.0 % and 28.3 %. The results showed the inhibition activity of TGO, TGD, or ox-TG on the cells was in a time-dependent manner. Our result is in agreement with Cao and co-workers findings [20]. They elucidated that there was significant inhibition activity of TGD on the cells at 50 μg/mL (*p <* 0.05), while it was 12.5 μg/mL (*p <* 0.05) for TGO on macrophages viability, which indicated that TGO was more reactive than TGD.

### Cell cycles analysis of TGO, TGD, or ox-TG on HepG2 cells

The propidium iodide (PI), a fluorochrome, staining is widely used for the evaluation of apoptosis. DNA fragmentation is one of the features in apoptotic cells. The nuclear DNA could be identified and quantified *via* the bind of PI and DNA. Thus, apoptotic cells could be detected by PI flow cytometry assay, which would display a sub-G1 (or hypodiploid) DNA profile because of the loss of DNA content.

**Fig. 2:**
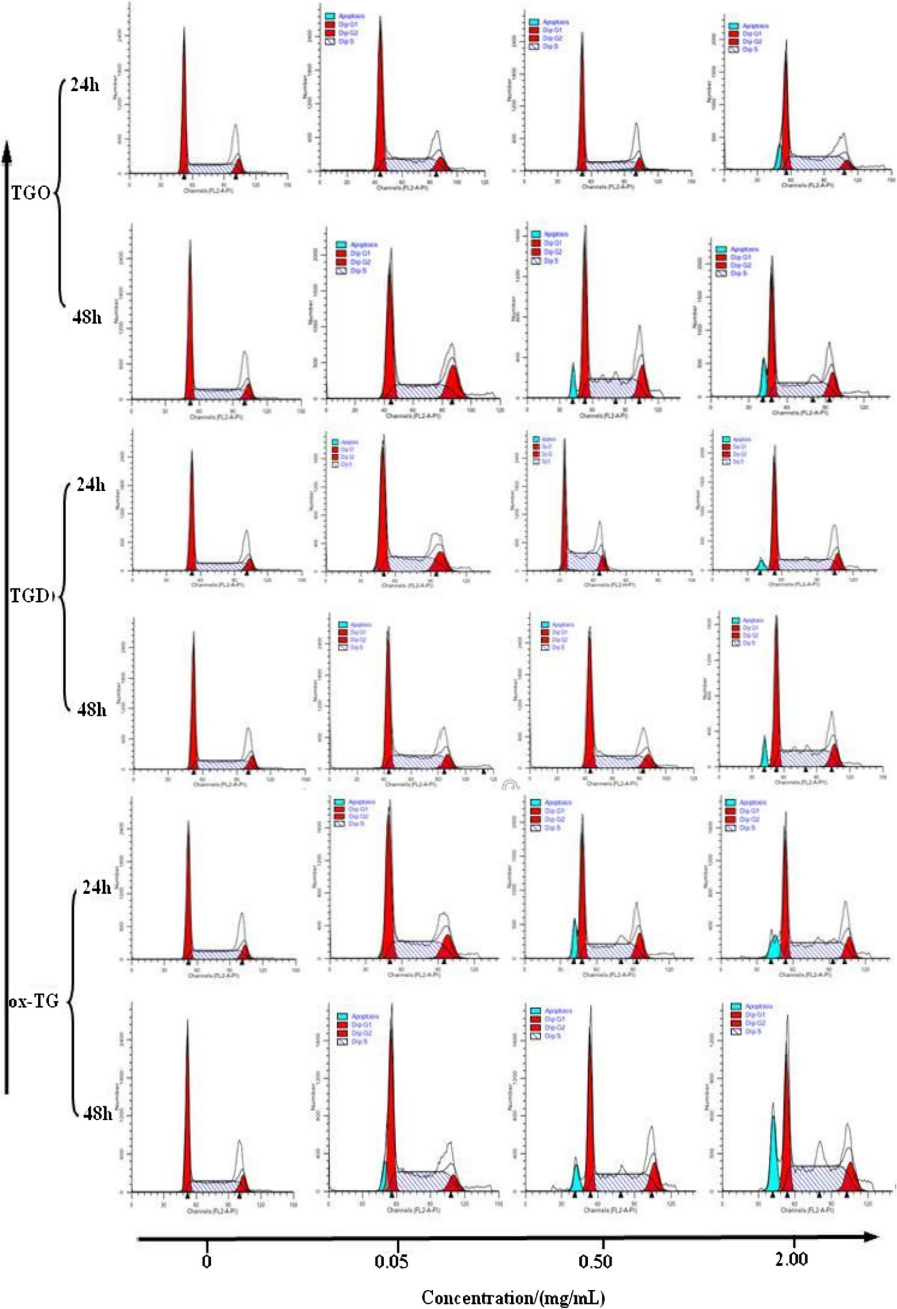
The cycle diagram of HepG_2_ cells incubated with TGO, TGD, and ox-TG

As shown in the fluorescence histogram (Fig. 2), the cells in negative control group display a sharp diploid DNA profile either with 24 h- or 48 h-incubation, and no apoptosis of cell appeared during 48 h-incubation. However, the exposure of the cells to TGO, TGD, or ox-TG led to the hypodiploid cells in a concentration- and time-dependent manner, which indicated that TGO, TGD, and ox-TG were deleterious to HepG2 cells. Ox-TG treatment induced apoptosis of the cells at 0.05 mg/ml after 48 h. While, sub-G1 cells were also detected in the HepG2 cells being exposed to TGO with a concentration of 0.5 mg/mL for 48 h. With a concentration of 2.0 mg/mL, all of TGO-, TGD-, and ox-TG-treated groups were detected apoptotic cells after 24 h incubation. It is obvious that apoptotic cells elevated with the increase of TGO, TGD and ox-TG concentration and time. Among them, ox-TG showed the most serious deleterious on cell apoptotic. Another in vitro study conducted by Ute Stemmer also illustrated that the oxidized lipids, specifically oxidized phospholipids, could induce apoptosis in macrophages [21].

Cell cycle analysis was performed to illustrate the effect of TGO, TGD, and ox-TG on cell proliferation (Table 1). The percentage of cells in S phase increased and those in G0/G1 phase decreased in HepG2 cells exposed to 0.05, 0.5 or 2.00 mg/mL ox-TG for 24 h/48 h. A similar concentration- and time-related increase was observed in TGO- and TGD-treated groups, which indicated that the inhibition of TGO, TGD, and ox-TG on HepG2 cells mainly occurred in S phase. As shown in Table 1, unexposed cells had 45.68 % of cells in G0/G1 phase and 41.67 % of cells in S phase after 48 h. However, 29.74 % of cells in G0/G1 phase and 57.60 % of those in S phase were detected when HepG2 cells were exposed to 2.00 mg/mL ox-TG for 48 h.

**Table 1:** The proportion of the HepG2 phase of G_0_/G_1_ cells and S in the total number of cells incubated with TGO, TGD and ox-TG

	Time (h)	24		48	
	Concentration (mg/mL)	G_0_/G_1_ (%)	S (%)	G_0_/G_1_ (%)	S (%)
Control	0.00	46.32 ± 1.69	41.05 ± 1.33	45.68 ± 0.61	41.67 ± 0.74
TGO	0.05	47.48 ± 1.41	38.93 ± 1.52	46.28 ± 0.15	43.47 ± 0.17
TGO	0.50	45.88 ± 0.64	44.74 ± 0.57	40.25 ± 0.83	49.37 ± 0.97
TGO	2.00	38.84 ± 0.18	49.42 ± 0.17	36.41 ± 0.24	54.54 ± 0.42
TGD	0.05	47.69 ± 1.59	42.95 ± 1.44	43.40 ± 0.56	42.96 ± 0.18
TGD	0.50	43.20 ± 0.10	47.62 ± 0.32	38.63 ± 0.78	50.00 ± 0.57
TGD	2.00	37.60 ± 0.08	50.49 ± 0.09	33.87 ± 0.85	57.68 ± 0.76
ox-TG	0.05	40.36 ± 1.35	49.42 ± 1.46	38.18 ± 0.32	50.49 ± 0.34
ox-TG	0.50	37.06 ± 0.63	49.69 ± 0.61	36.41 ± 0.79	51.55 ± 0.68
ox-TG	2.00	33.67 ± 0.27	54.92 ± 0.18	29.74 ± 0.35	57.60 ± 0.45

### Cell apoptosis analysis of TGO, TGD, or ox-TG on HepG2 cells

To confirm the apoptotic effects of TGO, TGD, and ox-TG on HepG2 cells, morphology of the cells was detected *via* flow cytometry. Apoptosis of the cells results in nucleus and cytoplasm fragmentation, leading to debris production, which would contribute to different scattering characteristics. Forward scatter (FSC) can be used to determine the size of the cells, and side scatter (SSC) represents the granularity of the cells. In the early stage of apoptosis, FSC reduces dramatically, and SSC either increases or maintains constant. However, both FSC and SSC decrease in the late stage of apoptosis. Based on the signal of FSC and SSC, apoptotic rate was determined.

It is obvious that TGO, TGD, and ox-TG could induce apoptosis of HepG2 cells in a concentration- and time-dependent manner (Table 2). As the concentration of TGO, TGD, or ox-TG increased, the apoptotic rate of the cells induced by TGO, TGD, and ox-TG all elevated dramatically (*P* < 0.05). At the incubation time of 24 h, apoptotic rate of cell induced by TGO could improve from 1.03 – 9.25 % when the concentration increased from 0.05 mg/ml to 2.00 mg/ml, while it improved from 0.83 – 4.89 % for TGD and it improved from 4.46 – 14.54 % for ox-TG. Meanwhile, an increased incubation time could also contribute to the raise of apoptotic rate of the cells. At the concentration of 2.00 mg/ml, apoptotic rate of cell induced by TGO could improve from 9.25 – 10.12 % when the incubation time increased from 24 – 48 h, while it improved from 4.89 – 6.02 % for TGD and it improved from 14.54 – 16.05 % for ox-TG. The polar compounds-induced apoptosis activity in HepG2 decreased in the order of ox-TG > TGO > TGD, and ox-TG has the strongest apoptosis activity compared to TGO and TGD.

**Table 2:** Apoptosis rate of HepG2 cells incubated with TGO, TGD and ox-TG

	Time (h)	24	48
	Concentration (mg/mL)	Apoptosis rate (%)	Apoptosis rate (%)
Control	0.00	0.05	0.07
TGO	0.05	1.03	1.69
TGO	0.50	5.81	6.13
TGO	2.00	9.25	10.12
TGD	0.05	0.83	1.31
TGD	0.50	2.81	3.91
TGD	2.00	4.89	6.02
ox-TG	0.05	4.46	5.64
ox-TG	0.50	10.32	12.21
ox-TG	2.00	14.54	16.05

The above experiment was carried out on HepG2 and the future researches should further investigate the effect of the different polar compounds-induced apoptosis activity on normal cells, including cell proliferation, apoptosis and possible genetic damage. The mechanism of polar compounds-induced apoptosis of enhancing the lipid peroxides in the cells, depleting antioxidants in the cells, enhancing genetic damage or a combination of these actions should also be addressed.

## Conclusion

Cell viability assay showed that among the frying oil polar compounds of ox-TG, TGO or TGD, ox-TG was the strongest inhibiter for HepG2 cells viability. The inhibition rate on HepG2 decreased in the order of ox-TG > TGO > TGD. Moreover, the inhibition activities of TGO, TGD, or ox-TG on the HepG2 cells were in a concentration- and time-dependent manner.

The exposure of HepG2 cells to TGO, TGD, or ox-TG could lead to the hypodiploid cells in a dose- and time-dependent manner, which indicated that TGO, TGD, and ox-TG all contributed to the apoptosis of HepG2 cells. Among them, ox-TG showed the most serious deleterious on cell apoptotic.

Cell cycle analysis showed that the percentage of cells in S phase increased and G0/G1 phase decreased in HepG2 cells exposed TGO, TGD, and ox-TG to 0.05, 0.5 or 2.00 mg/mL, which indicated the inhibition of TGO, TGD, and ox-TG on HepG2 cells mainly occurred in S phase.
